# Editorial: Drug discovery from herbal medicines/polypeptides for neurological diseases

**DOI:** 10.3389/fphar.2025.1615452

**Published:** 2025-06-02

**Authors:** Qi Liang, Jianfeng Zhang, Li Lin, Junfeng Wang

**Affiliations:** ^1^ Shaanxi Institute for Food and Drug Control, Xi’an, Shaanxi, China; ^2^ Department of Pharmacy, Eighth Hospital of Xi’an City, Xi’an, Shaanxi, China; ^3^ Department of Orthopedic Surgery, Tangdu Hospital, Air Force Medical University, Xi’an, Shaanxi, China; ^4^ Gordon Center for Medical Imaging, Massachusetts General Hospital & Department of Radiology, Harvard Medical School, Boston, MA, United States

**Keywords:** TCM, herbal medicines, neurological diseases, drug discovery, polypeptides

## 1 Introduction

The quest for effective treatments for neurological diseases—ranging from Alzheimer’s disease (AD) (Bai et al., 2024) and depression to stroke and spinal cord injury (Hu et al., 2023)—has driven researchers to explore unconventional therapeutic avenues. Among these, herbal medicine and polypeptide-based therapies stand out for their unique mechanisms and historical validation (Gong et al., 2024). This editorial synthesizes insights from 14 recent studies, highlighting their contributions to advancing drug discovery for neurological disorders and addressing the challenges of integrating traditional knowledge with modern pharmacology.

## 2 The renaissance of herbal medicine in neuroprotection

### 2.1 Alzheimer’s disease: targeting amyloid and tau pathology

Two studies exemplify the potential of plant-derived compounds in combating AD (Aili et al., 2023). The first (DOI: 10.3389/fphar.2025.1500955) investigates the biosynthesis of neuroactive alkaloids, such as Huperzine A and Galantamine. Both discoveries not only deepen our understanding of plant metabolism but also offer practical pathways for the sustainable production of crucial AD treatments. Complementing this, the second study (DOI: 10.3389/fphar.2025.1497861) explores natural inhibitors of glycogen synthase kinase-3β (GSK-3β), a key enzyme driving tau hyperphosphorylation. Curcumin derivatives from *Curcuma longa L.* and hesperetin from *Citrus × aurantium L.* were shown to reduce tau pathology in transgenic mouse models, highlighting GSK-3β as a pivotal target for multi-herbal interventions.

### 2.2 Depression and neuroplasticity: restoring balance

Depression, linked to impaired neuroplasticity, is addressed by two groundbreaking works (Dai et al., 2024). A review of traditional Chinese medicine (TCM) (DOI: 10.3389/fphar.2024.1426769) identifies compounds like gastrodin and saikosaponins, which upregulate BDNF expression and promote dendritic spine formation in the prefrontal cortex. Meanwhile, a meta-analysis of plant polysaccharides (DOI: 10.3389/fphar.2024.1348019) reveals their role in modulating the gut-brain axis. Polysaccharides from Astragalus and Rehmannia enhance serotonin synthesis by restoring gut microbiota diversity, offering a novel mechanism for antidepressant effects.

### 2.3 Ischemic injury and neurorepair

Cerebral ischemia-reperfusion injury, a major cause of stroke disability, is tackled by studies on apigenin (DOI: 10.3389/fphar.2024.1362301) and Guipi Wan (DOI: 10.3389/fphar.2024.1346226). Apigenin, a flavonoid in *Matricaria chamomilla L.*, was found to enhance DNA repair via PARP-1 activation, reducing infarct volume in rodent models. Guipi Wan, a TCM formulation, attenuated oxidative stress by regulating the Nrf2/HO-1 pathway, underscoring the value of multi-herbal synergies.

## 3 Polypeptides and bioactive alkaloids: precision tools for neural repair

### 3.1 Unlocking alkaloid potential

*Uncaria rhynchophylla (Miq.) Miq.* (DOI: 10.3389/fphar.2024.1436481). exemplifies the therapeutic promise of alkaloids. Its rhynchophylline and isorhynchophylline demonstrated NMDA receptor antagonism, reducing glutamate excitotoxicity in Parkinson’s models. Similarly, berberrubine from *Berberis vulgaris L.* (DOI: 10.3389/fphar.2024.1496917) protected against cisplatin-induced ototoxicity by upregulating folate biosynthesis enzymes, preserving cochlear hair cells.

### 3.2 Retinal and spinal cord protection

Innovative approaches for ocular and spinal disorders are emerging (Vargova et al., 2021). *Lycium ruthenicum Murray* extract (DOI: 10.3389/fphar.2024.1404119), rich in anthocyanins, preserved retinal ganglion cells in glaucoma models by inhibiting caspase-3-mediated apoptosis. For spinal cord injury, Erxian decoction metabolites (DOI: 10.3389/fphar.2024.1339956) binding PRAS40—a regulator of mTOR—enhanced axonal regeneration by modulating autophagy, achieving functional recovery in rats to some extent.

## 4 Clinical translation: bridging evidence and practice

### 4.1 Evaluating herbal formulations

The efficacy of standardized herbal mixtures (Hao et al., 2023) is exemplified by a blend of *Centella asiatica (L.) Urb., Echinacea purpurea (L.) Moench*, and *Zingiber officinale Roscoe* (DOI: 10.3389/fphar.2024.1439811), which normalized cortisol levels and restored dopaminergic signaling in stress-induced depression. Xingnaojing injection (DOI: 10.3389/fphar.2024.1411026), a TCM-derived neuroprotective agent, showed a significant reduction in post-hemorrhagic stroke edema in a meta-analysis of the patients, though heterogeneity in trial design calls for stricter standardization.

### 4.2 Antrodia camphorata: a fungal frontier

The *Zingiber officinale Roscoe* (DOI: 10.3389/fphar.2024.1372110) offers triterpenoids and polysaccharides that inhibit neuroinflammation via microglial TLR4 suppression. Its potential in treating multiple sclerosis is being explored in Phase II trials, with preliminary data showing an obvious reduction in relapse rates.

## 5 Challenges and future directions

Despite progress, critical hurdles remain:

1) Standardization: Batch variability in herbal extracts undermines reproducibility. This issue is expected to be resolved through the implementation of blockchain tracking with GACP, HPLC-MS fingerprint recognition, and reference standards.
2) Bioavailability: Previous studies have improved the bioavailability of natural products through nanocapsules (Amante et al., 2022), prodrug design (Beaumont et al., 2022), and synergistic formulations (Sharma et al., 2023).
3) Mechanistic Complexity: Herbal polypharmacology complicates target identification. Traditionally, network pharmacology contributed to this identification. Nowadays, AI-driven docking (as used in DOI: 10.3389/fphar.2024.1339956) is playing an increasingly important role in elucidating multi-objective effects.

## 6 Future research should prioritize

To advance herbal medicine into the era of precision and reproducibility, future research must prioritize three interconnected domains: multi-omics integration, sustainable bioengineering, and hybrid therapeutic trials. These priorities address critical gaps in standardization, scalability, and mechanistic validation while aligning with global demands for personalized and eco-conscious healthcare solutions.

### 6.1 Omics integration: decoding bioactive signatures

Herbal extracts’ complexity demands metabolomics (e.g., UPLC-QTOF-MS) to map bioactive markers linked to clinical outcomes. AI-driven platforms can integrate pharmacometabolomics and gut microbiome interactions. Standardizing protocols and addressing data heterogeneity remain challenges.

### 6.2 Sustainable sourcing: CRISPR-engineered plants

CRISPR editing resolves supply-chain instability and phytochemical variability through high-yield strains, precision chemotypes, climate resilience, and biosafety measures.

### 6.3 Hybrid trials: herbal-polypeptide synergies

Previous work proved that polypeptide such as BDNF-mimetic peptides was benifit for maintaining mitochondrial quality control (Ahuja et al., 2022). As such, the synergies of BDNF-mimetic peptides and natural products may be an alternatitive for neuroprotection.

## 7 Conclusion

The studies in this Research Topic illuminate a path forward where traditional herbal wisdom and cutting-edge polypeptide engineering converge to address neurological diseases. From alkaloids that recalibrate neurotransmitter systems to polysaccharides that heal the gut-brain axis, these therapies exemplify the power of nature-inspired innovation. However, their success hinges on resolving standardization, mechanistic clarity, and regulatory alignment. By fostering interdisciplinary collaboration—ethnobotanists, pharmacologists, and data scientists—we can transform these ancient remedies into the next-generation of neurological therapeutics, ensuring they meet the rigor of modern medicine while preserving their holistic essence. In this synergy of old and new lies the promise of healing some of humanity’s most complex disorders.

The convergence of herbal wisdom and polypeptide engineering presents groundbreaking potential for neurological therapeutics, yet critical methodological and translational challenges must be systematically addressed:

(1) Decoding Polypharmacological Synergy: Conventional reductionist approaches fail to capture the dynamic multi-target interactions of herbal compounds. Advanced methodologies should integrate multi-omics network modeling combining single-cell transcriptomics, metabolic flux analysis, and AI-enhanced molecular dynamics simulations.
(2) Bioinspired Peptide Delivery Optimization: Next-generation platforms are required to overcome the delivery barriers.
(3) Precision Standardization Systems: Although the blockchain-based herbal traceability platform and UPC^2^ chromatography integration have achieved dynamic monitoring of bioactive phytochemicals, the development of new technologies still needs to be emphasized.
